# Embodied Action Improves Cognition in Children: Evidence from a Study Based on Piagetian Conservation Tasks

**DOI:** 10.3389/fpsyg.2016.00393

**Published:** 2016-03-21

**Authors:** Mariana Lozada, Natalia Carro

**Affiliations:** INIBIOMA-CONICET, Universidad Nacional del ComahueBariloche, Argentina

**Keywords:** embodied cognition, enaction, piagetian conservation tasks, agency, children

## Abstract

Converging evidence highlights the relevance of embodied cognition in learning processes. In this study we evaluate whether embodied action (enaction) improves cognitive understanding in children. Using the Piagetian conservation tasks in 6–7 year olds, we analyzed quantity conservation conceptualization in children who were active participants in the transformation process and compared these results to those of children who were mere observers of an adult's demonstration (as traditionally conducted). The investigation was performed with 105 first-graders. Conservation tasks were demonstrated to half the children, while the other half actively carried out the transformation of matter. Our findings showed that active manipulation of the material helped children recognize quantity invariance in a higher proportion than when the demonstration was only observed. That is, their enactive experience enabled them to comprehend conservation phenomena more easily than if they were merely passive observers. The outcome of this research thus emphasizes how active participation benefits cognitive processes in learning contexts, promoting autonomy, and agency during childhood.

## Introduction

Numerous studies have emphasized the importance of embodied action in cognitive processing both in children and adults (e.g., Varela et al., 1991; Barsalou, 1999, 2008; Lakoff and Johnson, 1999; Thompson and Varela, 2001; Sommerville et al., 2005; Johnson and Rohrer, 2007; Glenberg, 2010; Witherington and Heying, 2013). The embodied cognition theory considers cognition as lived, enacted and closely intertwined with dynamic contexts (Varela, 2000; Thompson, 2007). This approach, which prioritizes the role of agency, considers cognition to be a consequence of active manipulation (e.g., Varela, 1999; Di Paolo et al., 2010). Thus, cognition results from sensory-motor experience, where action influences perception and vice versa, indicating that perception and action are inseparable (Prinz, 1990, 1997; Varela et al., 1991; Thelen and Smith, 1994; Jeannerod, 2001; Wilson, 2002; Rizzolatti and Craighero, 2004; Witherington, 2007, 2011; Fischer and Zwaan, 2008; Di Paolo et al., 2010; Di Paolo and De Jaegher, 2012; Anderson et al., 2013). In line with this, it has been stated that conceptual knowledge is embodied, and therefore grounded in sensory-motor systems (Gallese and Lakoff, 2005). To sum up, the embodied approach considers perception, action and cognition as tightly linked, and that previous sensorimotor experiences are seen as the basis of knowledge. It is therefore highly likely that first-person action improves conceptual understanding. In this investigation we evaluate how action promotes conceptual understanding.

One precursor of this view was Jean Piaget, who proposed that knowledge is linked to experience and demonstrated the crucial role of recurrent sensorimotor activity in developmental cognitive processes (Piaget, 1952). Piaget's huge contribution to child development showed that cognition is grounded in concrete activity, a theory he developed by studying how children shape their world throughout ontogeny through sensorimotor action (e.g., Piaget, 1952, 1971). Amongst his multiple contributions, Piaget found that understanding of the invariance principle, i.e., the logical concept of quantity conservation, is a developmental process that occurs between 5 and 8 years of age (Piaget, 1965, 1971). Piaget and other investigators applied a series of tasks in which children observed, then evaluated, whether a certain quantity remained the same when changes in visual appearance were introduced, even though nothing was added or removed (e.g., Piaget, 1971; Inhelder et al., 1974; McGarrigle and Donaldson, 1974; Wilkening, 1979; Alibali and Kita, 2010; Goldin-Meadow and Beilock, 2010; Ping and Goldin-Meadow, 2010). Thus, children's ability to understand conservation despite the apparent transformation of number, matter, or liquid quantity was analyzed. These conservation tasks are ideal for the evaluation of cognitive development, given that they are controllable, replicable and easy to apply in different cultures and socio-economic contexts. In addition, the results are easily measurable and these tests are therefore highly suitable for the assessment of the role of action in children's comprehension. Tasks consisted of showing a child an initial stage (e.g., pouring the same amount of liquid into two identical containers), then a transformation process demonstration (e.g., pouring the same amount of liquid into two different containers, so that quantities appeared to be different), and finally the child was asked whether the amount was the same as before, or different. The transformation processes involved the researcher, for example, flattening a ball of clay, stretching out a row of coins, or transferring a certain amount of liquid into a narrower or wider container.

According to Piaget (1965) and other researchers (e.g., Smedslund, 1968; Siegler, 1981; Alibali and Kita, 2010; Goldin-Meadow and Beilock, 2010; Ping and Goldin-Meadow, 2010; Houdé et al., 2011) children under 5–7 years old have difficulty understanding the conservation principle, whereas children older than 7–8 are generally capable of realizing that number, length, volume of liquid, and matter remain unchanged in spite of changes in form. Therefore, for the above situations, three developmental stages are expected to exist within the 5–8 age range: children who consistently recognize the conservation principle (Total Conservers, TC), those who partially recognize this concept (Partial Conservers, PC) and children who do not recognize it in any task (Non Conservers, NC). Children go through a transition stage of being “partial conservers” for liquid quantity, mass, number, and length (Church and Goldin-Meadow, 1986). Recognition of weight conservation occurs at 9–10 years of age, and 11–12 year-olds are able to understand volume conservation (Piaget, 1963). In relation to children's increased understanding of the conservation principle, a recent study on number conservation showed that certain neural networks associated with numerical and executive functions were not activated in 5–6 year-old children (non-conservers) whereas in children older than 9–10 (conservers) these networks were activated (Houdé et al., 2011). These authors suggested that the neural contribution of this bilateral parietofrontal network, associated with executive functions such as inhibitory control, plays a crucial role in the acquisition of number-conservation (Borst et al., 2012, 2013; Houdé and Borst, 2014).

Most previous studies on this subject analyzed the conservation principle in a situation where children observed the experimenter's demonstration (e.g., Piaget, 1965, 1971; Inhelder et al., 1974; McGarrigle and Donaldson, 1974; Wilkening, 1979; Goldin-Meadow and Beilock, 2010; Ping and Goldin-Meadow, 2010). These demonstrations precluded the possibility of enaction on the part of participants, who remained “passive,” i.e., they were not active agents. Following the enactive theory that action modifies perception, thus promoting further understanding, we considered that it would be of interest to explore whether active manipulation enhances conceptual understanding in children. In the present study we evaluate the role of hands-on experience in cognitive processing, hypothesizing that action will contribute to understanding of the conservation principle, analyzed by means of the well-known Piagetian conservation tasks, but with one major change: the addition of active manipulation. By giving participants the opportunity to handle the materials, we will be able to compare in a precise, concrete way, children's recognition of the conservation concept with and without the incorporation of action. That is, conceptual understanding will be assessed under two conditions: action and observation. Seven conservation tasks were devised using the Piagetian conservation tasks with 6–7 year olds, an age considered to be in the mid-range of this developmental process. We will test the hypothesis that when children are active participants in the transformation process, i.e., the act of transferring liquids, flattening a ball of plasticine, moving coins, or measuring strings with their own hands, conservation understanding is increased. Thus, we hypothesize that children's embodied action will help them to recognize quantity invariance in a higher proportion than children who merely observe the demonstration carried out by an experimenter.

## Material and methods

### Participants

The study was carried out with 105 first-graders from public and private schools in San Carlos de Bariloche, Argentina (54 girls, 51 boys). The age range of participants was 6–7 years (mean age: 6 years, 8 months ±0.6).

The participants were all in good health, and there were no significant differences in body mass index or socioeconomic level. Experiments were conducted according to the Helsinki declaration and approved by the Clinical Research Ethics Committee (CEIC) and by the Council of Education of Río Negro Province, Argentina. All procedures were carried out with the adequate understanding and written consent of parents and school authorities. In addition, the children gave their verbal consent for participation in the study.

### Procedure

During a normal school day, one child at a time participated in an experimental session of approximately 20–30 min. Two different procedures were followed, corresponding to an observation condition (*N* = 47, 25 girls, and 22 boys) and an action condition (*N* = 58, 29 girls and 29 boys). In each first grade, approximately half of the children were randomly assigned to each condition.

Children worked individually in a quiet room in the school. Each participant was told that he/she was going to play some games, and sat at a table, facing the researcher. The quantity conservation task materials were laid out on the table. Each session was recorded. We analyzed each answer per task per child, for both groups.

### Action and observation conditions

#### Action condition

In this condition the researcher, with speech and gestures, asked each child to carry out the seven Piagetian conservation tasks by themselves. The tasks were shown in the following order: Two liquid quantity tasks, two mass quantity tasks, a number task and two length tasks. Each task consisted of three stages: Sameness, changing and judgment stages.

1. In the liquid quantity task, the researcher instructed the child to pour the same amount of liquid into two identical containers in order to obtain equal quantities in each one. Each child was asked to make sure that both containers held the same amount of liquid. In the changing stage, the researcher instructed the child to pour the liquid from one of the two identical containers into a narrower one. Finally, in the judgment stage, the researcher asked the child if the quantities were the same or different, and why (“Can you say why you think they are the same/different?”).
2. The first stage of Task 1 was repeated but in the changing stage the researcher instructed the child to pour the liquid from one of the two identical containers into a much narrower one (narrower than in the changing stage of 1). In the judgment stage, the questions were repeated as for 1.
3. In the mass quantity task, the researcher instructed the child to form two identical balls (i.e., with the same amount of plasticine) and each child was asked to make sure the two forms had the same amount of matter. In the changing stage, the researcher instructed the child to roll the plasticine into a thinner, longer shape. In the judgment stage, the questions were repeated as for 1.
4. The first stage of Task 3 was repeated but in the changing stage the researcher instructed the child to flatten the ball and stretch it; in the judgment stage, the questions were repeated as for 1.
5. In the number task, the researcher instructed the child to arrange 20 coins in two rows of 10. Each child was asked to make sure the two rows had the same number of coins. In the changing stage, the researcher instructed the child to spread out the coins in the upper row. These instructions were accompanied with gestures for clarity. In the judgment stage, the questions were repeated as for 1.
6. In the length task, the researcher instructed the child to lay out two identical strings in a straight line in front of him/her, parallel to each other and to the edge of the table. Each child was asked to make sure the two strings were identical in length. In the changing stage, the researcher asked the child to move the upper string 20–30 cm to the right of its original location. In the judgment stage, the researcher asked the child if both strings were the same length and asked: “If an ant has to walk along both strings, will it travel the same distance on each? Will it take the same or a different number of steps?” and also asked why.
7. The first stage of Task 6 was repeated but in the changing stage the researcher instructed the child to form a curve with the upper string. These instructions were accompanied with gestures for clarity. Following this, the researcher asked questions as for 6.

#### Observation condition

In this condition, the child observed demonstrations of the seven Piagetian conservation tasks detailed above, in the same order as in the action condition and consisting of the same three stages, but in this case carried out by the researcher.

### Data analysis

#### Conservation judgments

We analyzed children's answers related to each task, i.e., if quantity remained the same or was different after the transformation process. If a child answered that quantity was the same, we considered this a conserver answer for that task. For both observation and action conditions, we analyzed each child's judgment. Children received no feedback on their answers, i.e., the experimenter did not comment on the children's responses.

#### Comparison of TC, PC, NC in each condition

If a child recognized conservation in all seven tasks, he/she was classified as a total conserver (TC). If a child recognized conservation in at least one task, but not in all, we considered him/her to be a partial conserver (PC), and if a child did not recognize conservation in any of the seven tasks, we considered him/her a non-conserver (NC).

### Statistical analysis

#### Conservation judgments

Using a chi square test (*p* < 0.05), we compared the proportion of conserver responses between the action and observation groups for each task category, taking tasks of a similar type together, i.e., liquid, mass, number, and length (Church and Goldin-Meadow, 1986). The conservation response was considered as accurate (score = 1) and non-conservation as error (score = 0).

#### Comparison of TC, PC, NC

We compared the proportion of children who recognized conservation in all 7 tasks (TC), those who recognized the conservation principle in at least one task but not all (PC) and those who did not recognize the conservation principle in any of the seven tasks (NC) in each group using a chi square test. The relative proportion of TC, PC and NC between groups was conducted by means of the difference test (*p* < 0.05).

## Results

**Figure 1:**
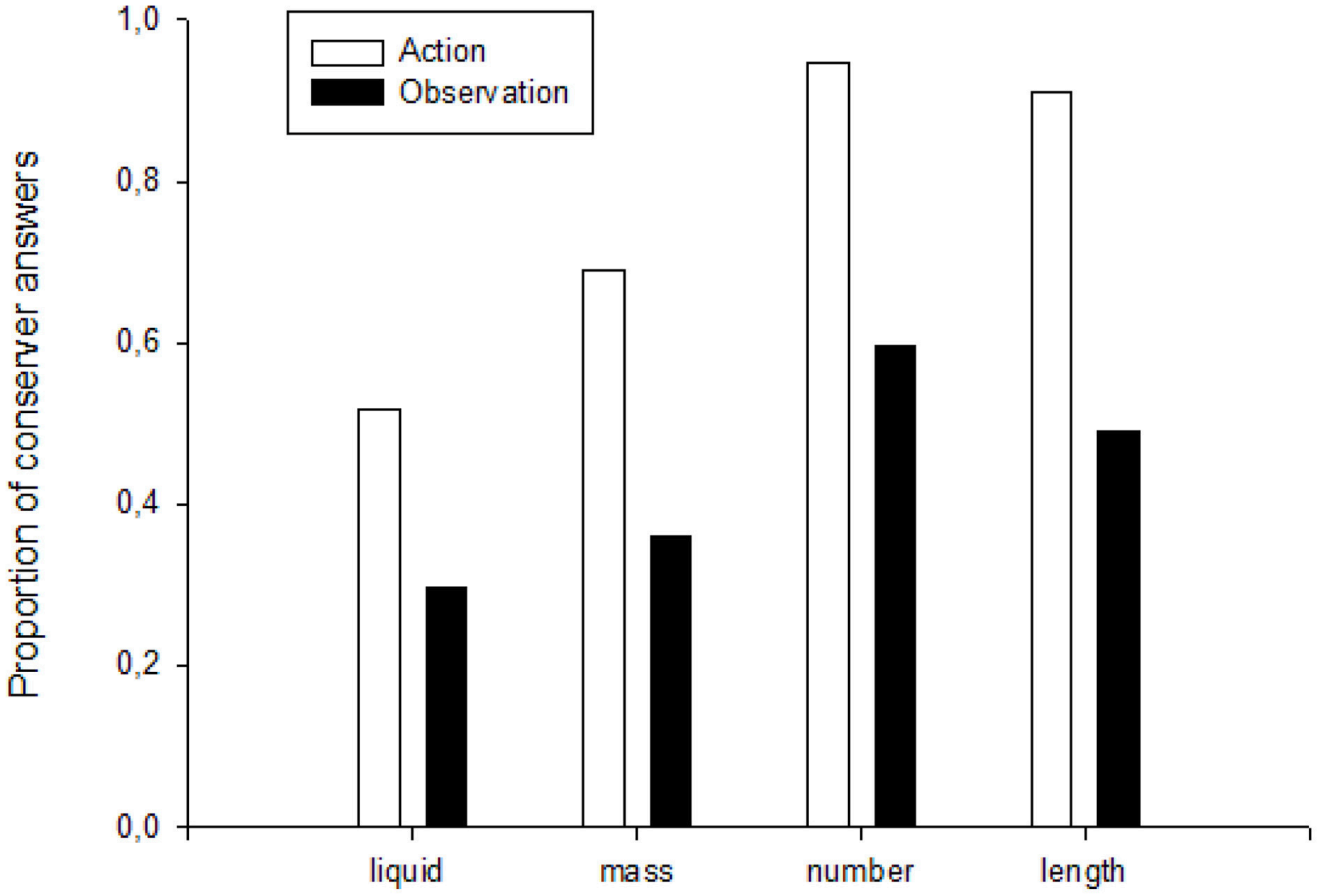
**Proportion of conserver answers in the action and observation groups for the four types of conservation tasks**.

When comparing the proportions of conservation responses for each task category (i.e., liquid, mass, length and number), for the action and observation groups, we found that in the liquid quantity tasks the proportion of conservation answers was significantly higher in the action group than in the observation one (*X*^2^ = 5.82, *p* = 0.0158, *df* = 1). That is, the act of transferring liquid by themselves helped the children recognize that liquid quantity was conserved despite the change in the container's shape. Similarly, a higher proportion of conservation answers was found in the action group during the mass tasks (*X*^2^ = 10.61, *p* = 0.0011, *df* = 1), i.e., the act of flattening or spreading out plasticine helped them recognize that there was no change in mass despite the transformation in shape. During the number task, the proportion of conservation answers was also higher for the action group (*X*^2^ = 20.08, *p* = 0.000, *df* = 1). Furthermore, the opportunity to handle the string during the length tasks led to a greater proportion of conservation answers in the action group than in the observation one (*X*^2^ = 23.21, *p* = 0.0000, *df* = 1) (Figure 1). Thus, in all tasks, a higher proportion of children who were given the opportunity to manipulate the materials showed a greater capacity for reasoning and discernment, which enabled their understanding of the conservation principle. As the study progressed, an overall increase in the proportion of conservation answers was observed in all tasks in both groups.

**Figure 2:**
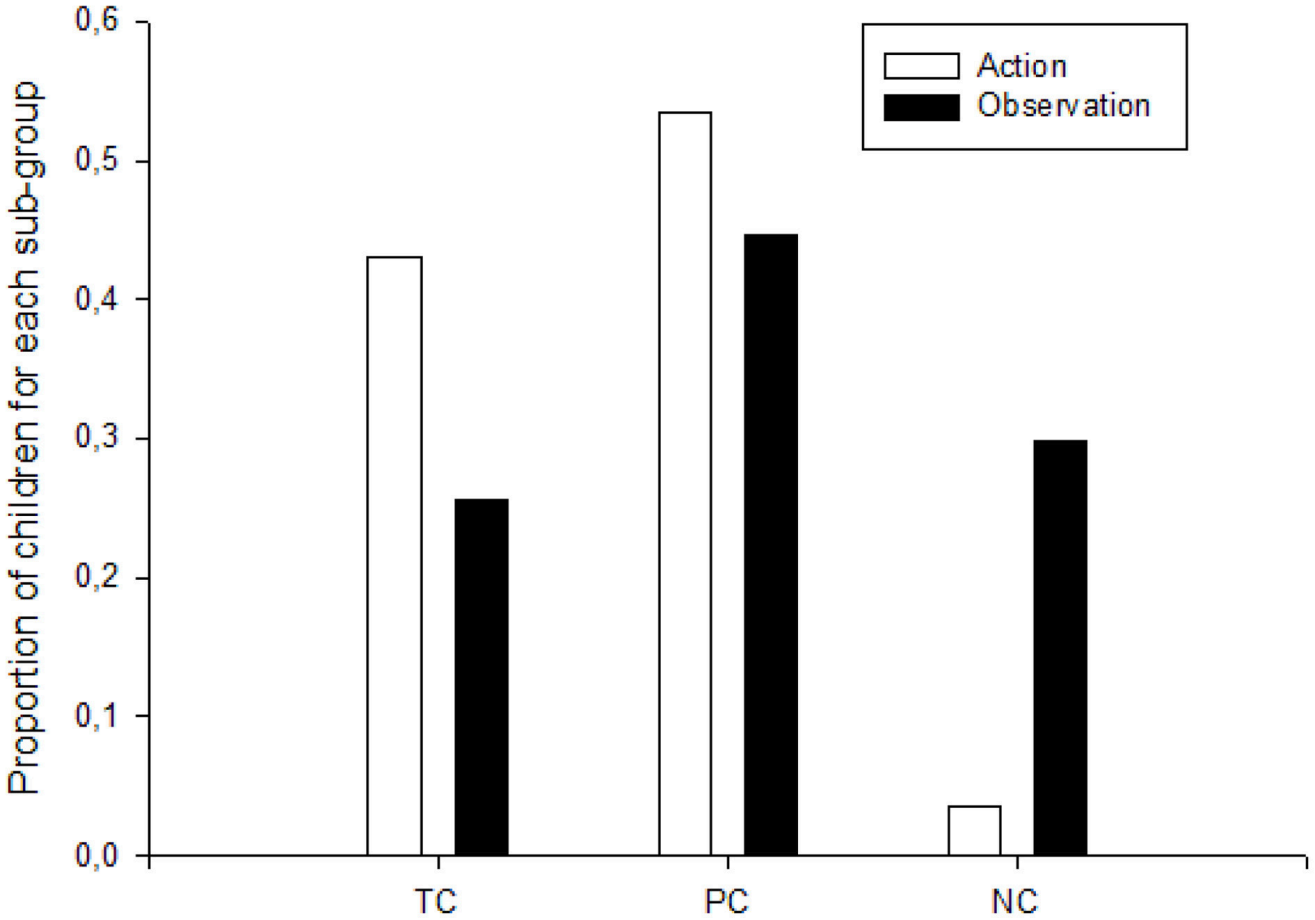
**Proportion of each subgroup (TC, PC, NC) in the action and observation groups**.

When comparing the relative proportion of children who recognized conservation in all tasks (TC), in at least one (PC), and in none (NC), we found significant differences between the three categories in the action group (*X*^2^ = 24.24, *p* = 0.0000, *df* = 2). Conversely, in the observation group no significant differences between the relative proportion of children who acknowledged conservation in all tasks, in at least one, and in none (*X*^2^ = 2.85, *p* = 0.24, *df* = 2). The action group holds a significantly higher proportion (*p* < 0.028) of children who recognized conservation in all tasks (43.10%) in comparison with the proportion found in the observation group (25.53%). In addition, in the action group we found a significantly lower proportion (*p* < 0.0001) of children who did not recognize conservation in any task (3.45%) compared with the proportion found in the observation group (29.79%). The extremely low number of non-conservers in the action group shows that when embodied action was allowed, most of the children were aware of conservation in at least one task in a higher proportion than when they were merely observers. The relative proportions of children who understood conservation in at least one task, but not in all, was similar in both groups (*p* = 0.21) (Figure 2); this fact could be associated with the high probability of responding correctly to one question out of six for the seven tasks. Therefore, we consider that the relevant finding is the higher proportion of TC found in the action group as well as the lower proportion of NC in this group.

## Discussion

The present study shows how active participation enhances cognitive processing in 6–7 year old children. Using the well-known Piagetian conservation tasks, we discerned that active manipulation, as opposed to mere observation of a demonstration carried out by a researcher, significantly increased understanding of the conservation principle. The fact that conservation performance was higher in the action group than in the observation one suggests that the experience of manipulating objects throughout the transformation processes facilitated recognition of the conservation concept. Thus, the experience of “doing” (i.e., being the agent) seems to favor conceptualization of the fact that liquid quantity, mass, number, and length can remain unaltered despite changes in appearance. Our findings contribute to the understanding of cognition as actively embodied, highlighting the impact of action in learning processes. These results suggest that education systems could benefit from the inclusion of embodied experiences in their teaching methods, as suggested in the past (e.g., Montessori, 1914; Dewey, 1920). Our research experimentally confirms the proposal of these well-known educators to incorporate first-hand experience in educational instruction (e.g., Montessori, 1914; Dewey, 1920).

This study aimed to confirm the important role of action in cognition. To this end, we chose to work with Piaget's conservation tasks, as they are well formulated and easily applicable. We sought to evaluate whether active manipulation influences children's cognitive processing, and therefore in our study children had the opportunity to perform the tasks, thus promoting agency and autonomy. The present work contributes to understanding of how children's cognitive abilities can be enhanced by active performance, favoring children's empowerment. Our findings are in line with recent studies that have shown how acting out can improve abstract comprehension of physics and mathematics (e.g., Glenberg et al., 2012; Nemirovsky et al., 2012; Núñez, 2012) and reading comprehension (e.g., Guan et al., 2013), highlighting how embodied experiences promote conceptual comprehension and rational and abstract thinking (Varela et al., 1991).

Our results agree with the embodied cognition theory which asserts that cognitive processes emerge from perception-action patterns, where action guides perception in local situations, which change, in turn, as a consequence of the child's action (e.g., Varela, 1999; Di Paolo et al., 2010). As proposed by the enactive theory, cognitive agents are not passive data collectors who model the world, but active participants who enact a world (Varela et al., 1991; Di Paolo and De Jaegher, 2012). Thus, the world is not something given from outside but something engaged in by doing; then, the world is “brought to life” by concrete handling (Varela, 2000). Along the same lines, a previous study comparing action and observation in conceptual categorization in adults found that action guides object categorization; the authors thus propose that categorization is based on sensorimotor experience (Iachini et al., 2008). In our investigation, the action and observation conditions also implied different sensorimotor experiences, suggesting that increased awareness of the invariance principle could be a result of the children's own activity, where feedback from their action may change their perception of the conservation phenomenon. It is likely that the different kinds of experience, i.e., observing or doing, could have led to different allocation of attention. That is, active manipulation could have helped increase attention to the transformation processes, promoting greater awareness and understanding. It has been proposed that concrete handling helps one focus, and so the present moment can be more salient when it involves concrete action (Varela, 2000). This is in line with previous studies which proposed that lack of attention to relevant quantitative relationships led young children to fail in conservation awareness (e.g., Trabasso and Bower, 1968; Gelman, 1969; Miller and Heller, 1976; Gelman and Baillargeon, 1983).

When analyzing each task performance, the results showed how children in the action group answered as conservers in all tasks in a higher proportion than in the observation group. The task sequence was randomly established and tasks were presented in the same order in both groups, i.e., children underwent the testing in identical conditions and were asked the same questions except for the opportunity to participate actively; the marked difference observed between groups, therefore, must have been due to a different cognitive process occurring while the action group performed the tasks. As the experiment progressed, in both groups a relative increase was observed in children's correct total conservation answers in all tasks except for the length one; this could indicate that generalization or learning transfer has occurred in both conditions. However, our objective was to compare performance under the action and observation conditions; that is, whether the act of “doing” or “observing” generated changes in the understanding of the conservation principle. To this end, the same sequence was followed for each participant and no counterbalancing of task evaluation was carried out. Future studies could further elucidate whether active manipulation of a certain conservation task can improve conservation awareness of a later one, and also evaluate the relative difficulty of each task.

As we worked with children whose age lay in the mid-range of the conservation invariance developmental process, it is significant that when comparing the proportion of total conservers, partial conservers, and non-conservers, we found a higher proportion of children in the action group who recognized the conservation principle in all tasks and a lower proportion of children who did not recognize quantity conservation in any task, as hypothesized. We worked with children of 6–7 years old, but previous research has shown that when an easier version of the task is applied, children show the conservation principle earlier in life (Wilkening, 1979); other studies which explored different ways of testing conservation principle allude to potential biases in Piagetian task methodology (e.g., McGarrigle and Donaldson, 1974) and also suggested that conservation conceptualization could occur at earlier ages. Nevertheless, the aim of our study is not to determine at what developmental age the conservation principle is acquired, but rather to compare its conceptualization in two clear-cut circumstances, one of which involved action on the part of children while the other did not.

Other research has demonstrated that children participating in these Piagetian tasks tend to explain the conservation concept with both gestures and speech; these embodied gestures can help manifest conceptual knowledge of conservation (e.g., Evans and Rubin, 1979; Church and Goldin-Meadow, 1986; Alibali and Kita, 2010). It has been found that gestures highlight perceptually present information (Alibali and Kita, 2010) and that gestures not only reflect unspoken thought but can also change thought in children (e.g., Goldin-Meadow, 2009; Goldin-Meadow and Beilock, 2010). These authors suggest that gestures provide a bridge between action and abstract thought. Furthermore, although our findings tie in well with these studies, the analysis of gestures constitutes a different approach to the relevance of action in cognitive processing. In a previous study researchers explored the use of training procedures between the pretesting and post-testing of conservation tasks in partial conservers. Results showed that the training, which involved manipulation, slightly improved posttest judgment (Church and Goldin-Meadow, 1986). In their investigation, the experimenter played a predominant role as he/she filled the two containers with the same quantity of liquid and then asked the child to transfer it to different containers. This fact could explain why they observed a much lower level of impact due to manipulation. In contrast, our research involved no training stage and the children were active agents of the entire process, from the beginning, without the active participation of an adult.

In our work, the fact that children's active experience during the transformation process was effective in facilitating conservation performance highlights the crucial role of agency. This could have an impact on education, given that, as stated by John Dewey, learning methods are generally external to children's existing capabilities, tending to lie beyond the reach of their experience. As he pointed out, “traditional teaching tends to impose from above and from outside adult standards, subject-matter and methods, and this imposition and external control oppose expression and cultivation of individuality” (Dewey, 1920). Similarly, the renowned educator Maria Montessori founded an integral pedagogy system based on the importance of a child's experience, maintaining that children learn when developing activities through the manipulation of objects (e.g., Montessori, 1914). To sum up, active participation during learning, therefore, connects children's experience more closely with cognitive processes, thus enriching educational practices while enhancing learning abilities As proposed by Jonas (1966), we actively maintain our dynamic identity by doing, i.e., our self-produced identity is based on action, and on perception-action patterns that enable constant interchange with the surroundings. The present study provides new evidence of how cognition emerges from experiential (enactive) processes, which contributes to the understanding of how embodied agency facilitates conceptual processing such as the quantitative conservation principle. The outcome of this research thus emphasizes how active participation benefits cognitive processes in learning contexts, promoting autonomy and agency during childhood.

## Author contribution

ML conceived, and designed experiments, analyzed data and contributed to the writing of the manuscript, drafting the work, final approval of the version to be published, and agreement to be accountable for all aspects of the work in ensuring that questions related to the accuracy or integrity of any part of the work are appropriately investigated and resolved. NC conducted experiments, collected data and contributed to the writing of the manuscript, drafting the work, final approval of the version to be published, and agreement to be accountable for all aspects of the work in ensuring that questions related to the accuracy or integrity of any part of the work are appropriately investigated and resolved

## Funding

This work was partially supported by Universidad Nacional del Comahue and INIBIOMA-CONICET.

### Conflict of interest statement

The authors declare that the research was conducted in the absence of any commercial or financial relationships that could be construed as a potential conflict of interest.
